# A Lightweight Convolutional Neural Network Based on Visual Attention for SAR Image Target Classification

**DOI:** 10.3390/s18093039

**Published:** 2018-09-11

**Authors:** Jiaqi Shao, Changwen Qu, Jianwei Li, Shujuan Peng

**Affiliations:** Naval Aviation University, Yantai 264001, China; 18153506607@163.com (J.S.); qcwwby@sohu.com (C.Q.); peng_shu_juan@163.com (S.P.)

**Keywords:** SAR, classification, convolutional neural network, visual attention, depthwise separable convolution, imbalance data

## Abstract

With the continuous development of the convolutional neural network (CNN) concept and other deep learning technologies, target recognition in Synthetic Aperture Radar (SAR) images has entered a new stage. At present, shallow CNNs with simple structure are mostly applied in SAR image target recognition, even though their feature extraction ability is limited to a large extent. What’s more, research on improving SAR image target recognition efficiency and imbalanced data processing is relatively scarce. Thus, a lightweight CNN model for target recognition in SAR image is designed in this paper. First, based on visual attention mechanism, the channel attention by-pass and spatial attention by-pass are introduced to the network to enhance the feature extraction ability. Then, the depthwise separable convolution is used to replace the standard convolution to reduce the computation cost and heighten the recognition efficiency. Finally, a new weighted distance measure loss function is introduced to weaken the adverse effect of data imbalance on the recognition accuracy of minority class. A series of recognition experiments based on two open data sets of MSTAR and OpenSARShip are implemented. Experimental results show that compared with four advanced networks recently proposed, our network can greatly diminish the model size and iteration time while guaranteeing the recognition accuracy, and it can effectively alleviate the adverse effects of data imbalance on recognition results.

## 1. Introduction

Synthetic aperture radar (SAR) is an active ground observation system that can be installed on aircraft, satellites, spaceships and other flight platforms. Compared with the optical and infrared observation methods, SAR can overcome the adverse effects of weather and perform dynamic observations of ground and ocean targets, so it has bright application prospects in the field of remote sensing. Compared with natural images, SAR images reflect the backscattering intensity of electromagnetic information, so specialist systems are needed to interpret them, but searching for targets of interest in the massive SAR images by humans is time-consuming and extremely difficult, which justifies the urgent need for SAR automatic target recognition (SAR-ATR) algorithms [1]. In the era of big data, there are tons of SAR image data waiting to be processed every day. Therefore, SAR-ATR requires not only high recognition accuracy, but also efficient data processing flows.

The traditional SAR image target recognition methods are mainly composed of independent steps such as preprocessing, feature extraction, recognition and classification. The feature extraction process usually needs scale invariant feature transform (SIFT) [2], histogram of oriented gradient (HOG) [3] and other algorithms to extract good distinguishing features to better complete the classification task. However, both the accuracy and efficiency of SAR image recognition are seriously restricted due to the complicated process and hand-designed features [4,5]. 

In 2012, the deep CNN [6] proposed by Krizhevsky et al. achieved the error rate considerably lower than the previous state of the art results in the ImageNet Large Scale Visual Recognition Challenge (ILSVRC), and CNN became of great interest to the academic community. Since then, many CNN models such as VGGNet [7], GoogLeNet [8], ResNet [9], DenseNet [10] and SENet [11], have been proposed and constantly challenged the computer’s limit of image cognitive ability. In the last ILSVRC competition in 2017, the Top-5 error rate of the Image Classification task reached 2.251%, exceeding the human recognition level.

The exciting progress of CNN in the field of computer vision (CV) has encouraged people to think about how to apply CNN to target recognition in SAR images, and many scholars have made intensive studies of this topic. Some papers used CNN to accomplish the SAR image target classification experiments on the Moving and Stationary Target Acquisition and Recognition (MSTAR) [12] public data set. The accuracy has gradually increased from 84.7% [1] to more than 99% [13,14], which is higher than that of SVM [15], Cond Gauss [16], AdaBoost [17] and so on. Ding et al. [4] investigated the capability of a CNN combined with three types of data augmentation operations in SAR target recognition. Issues such as translation of target, randomness of speckle noise in different observations, and lack of pose images in training data are intensive studied. Huang et al. [18] studied the influence of different optimization methods on the recognition results of SAR images in CNN model. Huang et al. [19] discussed the problem of SAR image recognition under limited labeled data. Bentes et al. [20] compared four CNN models [4,21,22,23] used in SAR-ATR in recent years, and put forward a multiple resolution input CNN model (CNN-MR). In order to improve the learning ability of the network, the SAR images are processed to different resolution slices in CNN-MR. The performance of the CNN-MR in the experiments indicating that the informative features make a significant contribution to obtain higher accuracy in CNN models, but such data preprocessing will bring extra work. As summarized in [24], while deep learning has become the main tool for tasks like detection in CV on RGB imagery, however, it has not yet had the same impact on remote sensing. As far as we know, many CNN models [1,4,14,22] designed for SAR image recognition are shallow networks, and only a few frontier technologies are utilized in the field of CV. We consider that the following three aspects can be further studied in the task of SAR image recognition with CNN:

1. Many shallow CNN models just consist of several convolution layers, pooling layers and an output layer. The interdependencies between channels and spaces of feature maps are often overlooked. How to improve the expressive ability of CNN and extract informative features through network designing is a valuable research direction.
2. Because the source of SAR image acquisition is greatly limited, some data sets are highly imbalanced. When the traditional machine learning classification method is applied to the imbalanced dataset, the classifier is biased to minority classes in order to improve the overall accuracy, and the classification performance is seriously affected. As far as we know, the problem of data imbalance in SAR image target recognition has not been paid enough attention in the current research yet.
3. The huge amount of parameters is an obstacle when CNN is applied in practice. In SAR image recognition, attention should also be paid to reducing network parameters and computation consumption while ensuring accuracy.

The human visual system (HVS) can automatically locate the salient regions in visual images. Inspired by the HVS mechanism, several attention models are proposed to better understand how the regions of interest (ROIs) are selected in images [25]. The visual attention mechanism has been wildly applied in many prediction tasks such as natural language processing (NLP) [26], image/video caption [27,28], image classification [11,29] etc. In SAR image recognition, Karine et al. [25] combined the SIFT method with a saliency attention model and built a new feature named multiple salient keypoints descriptors (MSKD). MSKD is not used on the whole SAR image, but only the target area. The recognition experiments for both ISAR and SAR images show that MSKD can achieve a significant advantage over SIFT, which indicates that the application of the visual attention mechanism in SAR image recognition is feasible.

SENet [11] is a CNN model based on visual attention mechanism. It uses a gating mechanism to model channel-wise relationships and enhances the representation power of modules throughout the networks [30]. The authors of SENet developed a series of SE blocks that integrate with ResNet [9], ResNext [31] and Inception-ResNet [32], respectively. Experimental results on the ImageNet dataset show that the introduction of SEblock can effectively reduce the error rate. In ILSVRC 2017, SENet won the first place in image classification competition, indicating its effectiveness. 

Depthwise separable convolution [33] is a kind of model compression technique that reduces the number of parameters and amount of computation used in convolutional operations while increasing representational efficiency [34]. It consists of a depthwise (DW) convolution, i.e., a spatial convolution performed independently over every channel of an input, followed by a pointwise (PW) convolution, i.e., a regular convolution with 1 × 1 kernel, projecting the channels computed by the DW convolution onto a new channel space. Depthwise separable convolution have been previously shown in Xception [33] to allow for image classification models that outperform similar networks with the same number of parameters, by making more efficient use of the parameters available for representation learning. Many state of the art CNN models such as MobileNets [35], ResNext [31], ShuffelNet [36], SqueezeNet [37] etc. also adopt depthwise separable convolutions to reduce model parameters and accelerate their calculations.

Data imbalance exists widely in practical applications, such as detecting sea surface oil pollution through satellite radar images [38], monitoring illegal trade in credit cards [39], and classifying medical data [40], etc. The general methods of dealing with imbalanced classification problems can be divided into two categories. The first one is data level methods including over-sampling and under-sampling [41,42,43]. The core idea of over-sampling is to randomly copy or expand the data of minority classes, but it easily leads to over fitting problems and deteriorates the generalization ability of the model. The under-sampling method balances the number of each class by removing part of the samples in the majority class, but it often losses some important data, which cause large offset or distortion in the decision boundary. The second is the algorithm level methods represented by the cost sensitive learning [44]. This method generally does not change the original distribution of the training data, but it gives different misclassification costs for different classes, i.e., the misclassification cost of a minority classes is higher than that of majority classes. The cost matrix in cost-sensitive learning is difficult to obtain directly from the data set and misclassification costs are often unknown [45,46]. Buda et al. [47] investigated the impact of class imbalance on the classification performance of CNNs and compared some frequently used methods. Experimental results indicate that over-sampling is almost universally effective in most situations where data imbalance occurs.

Inspired by SENet [11] and the extensive application of depthwise separable convolution, we consider applying them to SAR image recognition tasks. Based on the visual attention mechanism, we first designed a channel-wise and spatial attention block as the basic unit to construct our CNN model. Then, depthwise separable convolution wis utilized to replace the standard convolution in order to decrease network parameters and model size. We also use a new loss function named weighted distance measure (WDM) loss to reduce the influence of data imbalance on the accuracy. The main contributions of our work are:

1. Propose a lightweight CNN model based on visual attention mechanism for SAR image classification. The utilization of channel-wise and spatial attention mechanism can boost the representational power of network. Experiment on MSTAR [12] dataset indicate that compare with CNN model without visual attention mechanism (e.g., ResNet [9], Network in literature [23] and A-ConvNet [22]), our network achieves higher recognition accuracy. Meanwhile, the model parameters and calculation consumption are significantly reduced by using depthwise separable convolution.
2. A new WDM loss function is proposed to solve the data imbalance problem in the data set, and a comparative analysis is done of different ways to deal with the data imbalance problem. Experimental results of MSTAR [12] and OpenSARShip [48] indicate the new loss function has a good adaptability for the imbalanced data set.

The rest of this paper is organized as follows: Section 2 illustrates the key technologies used to build our lightweight CNN, including channel-wise and spatial attention, depthwise separable convolution and its implementation, and WDM loss function. Furthermore, the technical details of network construction and network topology are also given. Section 3 conducts a series of comparative experiments based on two open datasets, i.e., MSTAR [12] and OpenSARShip [48]. The performance of the proposed network is demonstrated, and how to choose the hyper-parameters is discussed. Section 4 summarizes our work and puts forward the future research.

## 2. Lightweight CNN Based on Visual Attention Mechanism

### 2.1. Channel-Wise and Spatial Attention

**Figure 1:**
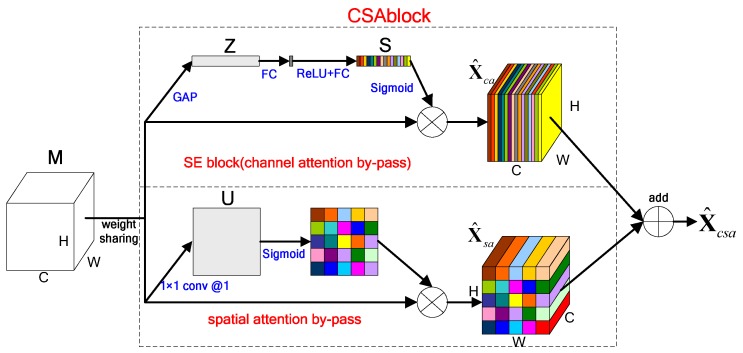
The structure of CSA block.

Convolution layers are the basic structure for CNNs. It learns filters that capturing local spatial features along all input channels, and generates feature maps of jointly encoding space and channel information. Squeeze and excitation (SE) block in [11] can be considered as a kind of channel-wise attention mechanism. It squeezes features along the spatial domain and reweights features along the channels. The structure of SE block is shown in the upper part of Figure 1. In SAR image target recognition, regions of interest are generally concentrated in a small area. Meanwhile, spatial information usually contains important features for accurate recognition, so it should also be used rationally. Inspired by SE block, we carry out similar operations on spatial, and introduce channel attention and spatial attention mechanisms on two parallel branches. Finally, we add the results from the two channels as the output. We call the above operation as channel-wise and spatial attention (CSA) mechanism, and the convolution unit is named CSA block, the structure of it is shown in Figure 1.

Suppose that the feature maps entering into CSA block is $M\in {\mathbb{R}}^{H\times W\times C}$, where H, W and C are the spatial height, width and channel depth respectively. In channel attention by-pass, M is represented as $M=\left[m_{1},m_{2},\cdots ,m_{C}\right]$, $m_{i}\in {\mathbb{R}}^{H\times W}$ represents the feature maps on each channel. Spatial squeeze is performed by global average pooling (GAP), a statistic $Z\in {\mathbb{R}}^{C}$ is generated by shrinking M through spatial dimensions H×W, where the c-th element of Z is calculated by:

(1) $$Z_{c}=F_{sq}(m_{c})=\frac{1}{W\times H}\sum_{i=1}^{W}\sum_{j=1}^{H}m_{c}(i,j)$$

After that, channel excitation is completed through a gating mechanism with sigmoid activation, vector Z is transformed to:

(2) $$S=F_{ex}\left(z,W\right)=\sigma (g(z,W))=\sigma (W_{2}\delta (W_{1}z))$$

In Equation (2), δ refers to the ReLU [49] function and σ represent sigmoid function, $W_{1}\in {\mathbb{R}}^{\frac{C}{r}\times C}$ and $W_{2}\in {\mathbb{R}}^{C\times \frac{C}{r}}$. The utilization of two fully-connected (FC) layers aims at limiting model complexity and aiding generalization, it is composed of a dimensionality reduction layer with parameters $W_{1}$ with reduction ratio r (we set it to be 8, and the parameter choice is discussed in Section 3.5), a ReLU function, and then a dimensionality-increasing layer with parameters $W_{2}$. The final output of the block is obtained by rescaling the transformation output M with the activations:

(3) $${\hat{x}}_{c}=F_{scale}(m_{c},S_{c})=S_{c}\cdot m_{c}$$

The output of channel attention by-pass is ${\hat{X}}_{ca}=[{\hat{x}}_{1},{\hat{x}}_{2},\cdots ,{\hat{x}}_{c}]\in {\mathbb{R}}^{H\times W\times C}({\hat{x}}_{c}\in {\mathbb{R}}^{H\times W})$, which represents the fusion features between channels.

In spatial attention by-pass, the input feature map is represented as $M=\left[m_{1,1},m_{1,2},\cdots ,m_{i,j},\cdots ,m_{H,W}\right]$, $m_{i,j}\in {\mathbb{R}}^{1\times 1\times C}$ with i∈{1,2,⋯,H} and j∈{1,2,⋯,W} represents the spatial features that contain all the channel information. Channel squeeze is performed by a 1 × 1 convolution kernel $K\in {\mathbb{R}}^{1\times 1\times C\times 1}$, generating a projection tensor $U\in {\mathbb{R}}^{H\times W}$, i.e., U=K*M. Each $U_{i,j}$ of U represents the linearly combination for all C channels in a spatial location (i,j). Similar to channel attention by-pass, we use the sigmoid function as nonlinear activation to complete spatial excitation. The output of spatial attention by-pass can be illustrated as:

(4) $${\hat{X}}_{sa}=[{\hat{x}}_{1,1},\cdots ,{\hat{x}}_{i,j},\cdots ,{\hat{x}}_{H,W}]$$

where, ${\hat{X}}_{sa}\in {\mathbb{R}}^{H\times W\times C}$ and ${\hat{x}}_{i,j}=\sigma (U_{i,j})\cdot m_{i,j}$.

Finally, we add the results of two by-passes (channel attention by-pass and spatial attention by-pass) to get the output of CSA block, i.e., ${\hat{X}}_{csa}={\hat{X}}_{ca}+{\hat{X}}_{sa}$. For the input feature map M, CSA block carries the future recalibrated through the channel and spatial, and it can enhance the expression ability of networks.

### 2.2. Depthwise Separable Convolution

**Figure 2:**
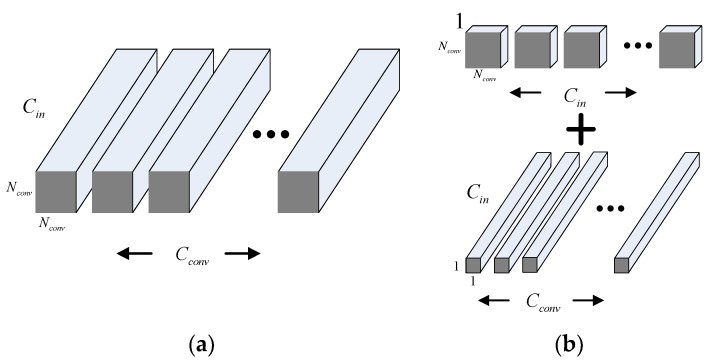
The distribution of convolution kernel (**a**) in standard convolution (**b**) in depthwise separable convolution.

In standard convolution, the channel of every kernel is the same as that of the current feature map $C_{in}$, and every channel is convoluted at the same time. The distribution of convolution kernel in standard convolution is shown in Figure 2a. Kernel size is $N_{conv}\times N_{conv}$, and the number is $C_{conv}$.

Depthwise separable convolution [33] uses DW convolution and 1 × 1 PW convolution to decompose convolution in channel level. DW refers to a convolution kernel that no longer carry out convolutions in all channels of the input image, but one input channel, i.e., one convolution kernel corresponds to one channel. After that, the PW convolution aggregates the multichannel output of the DW convolution layer to get the weight of the global response. The distribution of convolution kernel in depthwise separable convolution is shown in Figure 2b.

Through Figure 2a,b, we can make a brief analysis of the computation consumption of two convolution methods. The size of the input image is $N_{in}\times N_{in}$, with $C_{in}$ channels, the size of $C_{conv}$ kernels is $N_{conv}\times N_{conv}\times C_{in}$. In order to unify the output and input feature map in size, we assume the stride of convolution is 1, so the size of output features is $C_{conv}\times N_{in}\times N_{in}$. Ignoring the addition of features aggregation, the calculation amount required is $N_{in}\times N_{in}\times N_{conv}\times N_{conv}\times C_{in}\times C_{conv}$, the first two items are the size of the input image, and the other four are the space dimensions of the convolution kernel. When deep separable convolution is used, the calculation consumption of DW convolution is $N_{conv}\times N_{conv}\times C_{in}\times N_{in}\times N_{in}$ and the calculation consumption of PW convolution is $1\times 1\times C_{conv}\times C_{in}\times N_{in}\times N_{in}$. So we can get the ratio of calculation consumption of two convolutions is as follows:

(5) $$\begin{array}{l} \frac{N_{in}\times N_{in}\times N_{conv}\times N_{conv}\times C_{in}\times C_{conv}}{N_{conv}\times N_{conv}\times C_{in}\times N_{in}\times N_{in}+C_{conv}\times C_{in}\times N_{in}\times N_{in}}\\ =\frac{1}{C_{\mathrm{conv}}}+\frac{1}{N_{conv}^{2}} \end{array}$$

It can be seen from the above formula that the calculation consumption of deep separable convolution can be effectively reduced compared with the standard convolution, and the ratio of calculation consumption is only related to the number and size of the convolution kernel.

### 2.3. Weighted Distance Measure Loss Function

Imbalanced data have a great influence on the classification results, mainly because majority class data have more influence on classifiers than minority classes, so the classification boundaries are biased toward the majority classes. 

The common loss function in the field of machine learning, such as 0–1 loss function, log loss function and cross entropy loss function, have the same misclassification cost for all samples, and fail to be used directly in the problem of imbalance data classification. Therefore, new loss functions need to be designed for imbalanced data. On the other hand, the classification problem is a core problem in the research of pattern recognition, and a basic criterion in pattern recognition is to keep the inter class distance as large as possible and the intra class distance as small as possible. 

Through the above analysis, we can conclude that the loss function used for imbalanced data classification in CNN should meet the following requirements:

1. It should strengthen the influence of minority samples on training process, and avoid the submergence of minority samples by majority samples.
2. It should be well compatible with the CNN training process and can be calculated in batches.
3. It should enhance the inter class distance and reduce the intra class distance.

Contrastive loss [50] is used to solve the face recognition problem with long tailed distribution (which mean the number of categories is very large and not known during training, and the number of training samples for a single category is very small, and it can be regarded as a form of data imbalance.) data. This method requires a pair of samples as input, learning a similarity measure based on the input data, and then using the similarity measure to determine whether the two samples belong to one class and achieve the recognition results. The core idea of contrastive loss is put a small distance between similar samples, and large distance for dissimilar samples [51]. In generally, the purpose of the SAR image classification is not to judge whether the two slices belong to one class, but to identify what category the image belongs to. So contrastive loss function cannot be used directly. Even so, the thought of the contrastive loss function is of great reference. We combine the idea of contrastive loss and cost sensitive learning to design a weighted distance measure (WDM) loss function used for the problem of imbalanced data classification in CNN. The target of WDM loss function lies in two aspects, the first one is maximize the inter class distance and minimize the intra class distance, and the second one is make the samples of minority classes obtain a large compensation weight.

The WDM loss function can be expressed as the following form.

(6) $$L=\alpha L_{1}+\beta L_{2}$$

In Equation (6), $L_{1}$ represents intra class loss and $L_{2}$ represents inter class loss, α and β are loss weights of intra class and inter class respectively. α is set to $10^{-5}$ and β is set to $10^{-4}$.

We use w indicates the compensation weight, which is used to control the wrong cost of different classes. Supposing that there are N samples in M class totally, and the number of each class are arranged from large to small as $N_{1},N_{2},\cdots ,N_{m}$ (m=1,2,⋯,M). Then, compensation weight w can be expressed as $w=[w_{1},w_{2},\cdots ,w_{m}]=[N_{m},N_{m-1},\cdots ,N_{1}]/N$, which ensuring the minority classes can obtain a large compensation weight. $L_{1}$ can be further expressed as:

(7) $$L_{1}=\sum_{i\subseteq I}L_{1}^{i}w_{i}=\sum_{i\subseteq I}\frac{kw_{i}}{\sum_{j=1}^{k}\frac{1}{D_{j}}}$$

I represents the total classes of samples in a training batch, and $\sum_{j=1}^{k}\frac{1}{D_{j}}$ is defined as intra class distance measure. $D_{j}$ is the j-th longest Euclidean distance in one class. Suppose $x_{1}$ and $x_{2}$ are the two samples with the farthest distance in this class, $x_{3}$ and $x_{4}$ are the two samples with second-farthest distance, then there is $D_{1}={\left\|x_{1}-x_{2}\right\|}_{2}^{2}$, $D_{2}={\left\|x_{3}-x_{4}\right\|}_{2}^{2}$. k is a hyper-parameter (k is not a sensitive parameter, it can be set to 1 or 2, experience shows k=2 is a better choice.), showing the punishment strength of loss function to the intra class distance. The greater value of k means the greater the intensity of the punishment. Through Equation (7), we can see that the essence of intra class loss is the harmonic mean of the first k maximum distance measure.

$L_{2}$ is expressed as:

(8) $$\begin{array}{ll} L_{2} & =\max(m-D_{c},0)\\ & =\max(m-{\left\|x_{A}-x_{B}\right\|}_{2}^{2},0) \end{array}$$

In Equation (8), supposing that the inter class distance between the class *A* and *B* is the shortest. $D_{c}$ is defined as inter class distance measure, representing the shortest inter class. $x_{A}$ and $x_{B}$ denote the arithmetic mean of samples in class *A* and *B* after the last layer of CNN, which represents the center of the class characteristics. m is the threshold of loss function to punish the inter class distance. The smaller inter class distance will cause greater loss. We set m to $2\times 10^{4}$ and the results sensitive to it is discussed in Section 3.5.

In general, in the WDM loss function, we introduce the intra class distance measure $\sum_{j=1}^{k}\frac{1}{D_{j}}$ and the inter class distance measure $D_{c}$ to punish the problem that the intra class distance is too large and the inter class distance is too small. 

It should be explained that the contrastive loss function is based on a pair of samples, the optimization process is also aimed at a pair of samples and is a local optimization. The WDM loss function is based on a training batch, and the optimization process is also a global optimization for all kinds of samples.

### 2.4. Network Construction

#### 2.4.1. The Implementation of Depthwise Separable Convolution and CSA Block

When we build the network, we learn from the basic structure of ResNet [9]. When ResNet works, the core unit of it, i.e., the residual block first uses 1 × 1 convolution to compress the dimension of the input feature maps. Therefore, the subsequent 3 × 3 convolution will be completed on a lower data dimension. Finally, the data dimension will be restored by 1 × 1 convolution.

**Figure 3:**
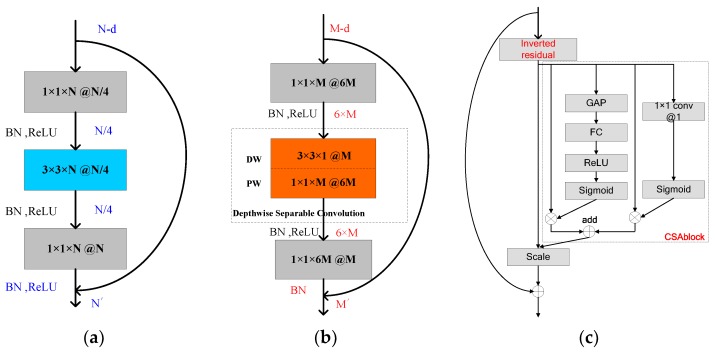
The structure of different basic blocks in CNNs (**a**) residual block (**b**) inverted residual block (**c**) inverted residual block with channel-wise and spatial attention (IR-CSA).

The structure of the residual block is shown in Figure 3a. In the whole process, data is compressed firstly and then expanded, so this structure is also called the bottleneck block. The data processing process in the bottleneck structure is shown in Table 1, t represents expansion factor and generally takes 0.25 in residual structure.

As introduced in Section 2.2, DW convolution uses a convolution kernel with one channel (as shown in Figure 2b), feature extraction capability has decreased compared with standard convolution. If depthwise separable convolution is directly used to replace the 3 × 3 standard convolution in the bottleneck structure, DW convolution will face the data of compressed dimension, which is more unfavorable for DW convolution to extract features. Therefore, refer to literature [52], we first enhance the dimension of data by a PW unit before using DW, that is, set expansion factor t to an integer bigger than 1 (we take t = 6, and the choice of it is discussed in Section 3.5) to make DW convolution reach a higher dimension of data. After that, a PW convolution is used to compress the data dimension. This structure is called inverted residual block, as shown in Figure 3b. In addition, related studies [52] also show that using non-linear layers in bottlenecks indeed hurts the performance by several percent, so in the inverted residual block, we remove the ReLU layer after the last 1 × 1 convolution to better retain the features.

Finally, the CSA block mentioned in Section 2.1 is added to the inverted residual structure to complete the fusion of the channel and the spatial features. The structure of the inverted residual block with channel-wise and spatial attention (IR-CSA) is shown in Figure 3c. We use IR-CSA structure as the basic convolution block to form the main structure of the CNN we propose. It is similar to ResNet [9] and many other networks, the main structure of the network is constructed by continuously stacking the basic convolution units.

#### 2.4.2. Network Topology

The main steps used in designing our network are summarized as below:

1. We use depthwise separable convolution instead of the 3 × 3 standard convolution in network to reduce the computational cost, and use the inverted residual block to improve the feature extraction ability of depthwise separable convolution.
2. The CSA block mentioned in Section 2.1 is introduced into the inverted residual structure to improve feature learning and fusion capabilities.
3. WDM loss function is applied to reduce the impact of imbalance data.
4. For the SAR image slice with input size 128 × 128, the larger size of convolution kernels are adopted to cope with the possible noise. We design the convolution kernel size in the first convolution layer to be 7 × 7, the performance of convolution kernels of different sizes under noise interference will be illustrated in Section 3.3.

The structure of lightweight network presented in this paper and ResNet50 [9] are shown in Table 2. Our network contains 12 IR-CSA blocks, and each IR-CSA block has 4 convolution layers and one CSA block. Similar to ResNet50, our network is also a 50-layer deep network, but its computing consumption is obviously less than it.

Only the main structure of the network is given in the Table 2. Other operations, such as batch normalization (BN), ReLU, etc. are not embodied in the table. The reduction of the size of the feature maps is achieved by setting the convolution step of 2.

## 3. Experiments and Discussion

### 3.1. Experimental Data Sets 

#### 3.1.1. MSTAR

**Figure 4:**
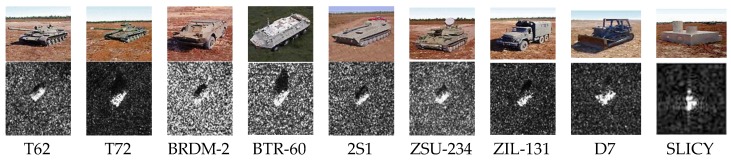
Examples of MSTAR dataset.

One of the datasets used in our work is part of MSTAR program [10], which is jointly sponsored by the U.S. Defense Advanced Research Projects Agency (DARPA) and Air Force Research Laboratory (AFRL). Hundreds of thousands of SAR images were collected containing ground targets, including different target types, aspect angles, depression angles, serial number, and articulation. SAR images in the dataset are gathered by the X-band SAR sensors in spotlight mode [25], with the resolution of 0.3 m × 0.3 m and $0\sim 360^{{}^{\circ}}$ azimuth coverage. Due to the lack of data, our dataset contains tanks: T62, T72; armored vehicles: BRDM2, BTR60; rocket launcher: 2S1; air defense unit: ZSU234; military trucks: ZIL131; bulldozer: D7; false target: SLICY nine types of targets, as shown in Figure 4. The lack of BMP2 and BTR70 also belong to the armored vehicles (the same as BRDM2 and BTR60), so influence on the cause can be ignored. Referring to the experiments in literature [22], 2770 images under $17^{{}^{\circ}}$ pitch angle were taken as training samples, and 2387 images were taken as testing samples under $15^{{}^{\circ}}$ pitch angle. 

Table 3 gives a list of training and testing data for 9 types of targets. From the table, we can see that the number of samples is relatively balanced, without significant difference.

#### 3.1.2. QpenSARShip

The OpenSARShip [48] is a new dataset built by Key Laboratory of Intelligent Sensing and Recognition, Shanghai Jiao Tong University, China. It contains more than ten thousands ship chips covering 17 AIS types from 41 Sentinel-1 SAR images with C-band [48]. These 41 Sentinel-1 SAR images are collected from five typical scenes because of their intense marine traffic: Shanghai Port (China), Shenzhen Port (China), Tianjin Port (China), Yokohama Port (Japan), and Singapore Port (Singapore). OpenSARShip provides two available products of the interferometric wide swath mode (IW): the single look complex (SLC) with 2.7 m × 22 m to 3.5 m × 22 resolution, and ground range detected (GRD) with 20 m × 20 m resolution [48].

**Figure 5:**
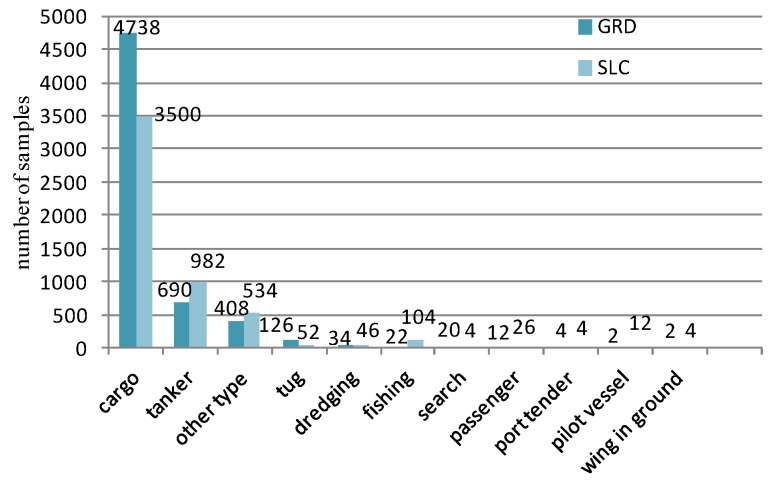
Statistical results of data set.

We classify the data set according to different polarizations and imaging modes. The distribution of the samples under GRD and SLC mode are shown in Figure 5. Each mode contains the same number of VH and VV polarization images, e.g., in the 4738 cargo slices of the GRD mode, there are 2369 images of VH and VV polarization, respectively. The data set includes cargo, tankers, tugs and other eleven types of ships.

It can be seen from Figure 5 that the class imbalance is quite serious in this data set. The cargo class accounts for more than 60% of the total in both modes. This imbalance may have a great impact on the recognition results. We divide the data into a training set and testing set in the proportion of 7:3, and eliminate the minority samples that are not enough to build the training and testing set. The data we used in experiments is shown in Table 4.

In [48], the authors completed a series of SAR image recognition experiments under VH and VV polarization, but the mode of the SAR images (GRD or SLC) was not clarified. In order to study the effects of different polarizations and modes on the recognition results we conduct a series of prior classification experiments under our network with different polarizations and imaging modes (i.e., GRD mode with VH polarization, GRD mode with VV polarization and SLC mode with VH polarization) SAR images.

Compared with the overall accuracy, we can clearly understand the recognition result of each class through the confusion matrix, and avoid the influence of the high recognition accuracy of majority classes on the overall recognition accuracy. Therefore, we use the confusion matrix as the evaluation index in this place and the subsequent experiments in Section 3.4. The results of prior classification experiments are shown in Table 5, Table 6 and Table 7.

From Table 5, Table 6 and Table 7, we can see the total recognition accuracy in three groups are all about 78%, and the *P* of the majority class (cargo) is significantly higher than that of the minority classes. Experimental result indicates that polarizations and imaging modes have no significant effect on the recognition results, but data imbalance has an obvious influence on it, so we only utilize SAR images under GRD mode with VH polarization for the subsequent experiments in Section 3.4.

### 3.2. Experimental Environment and Configuration

Most CNN models (including our network) require input images of the same size. Meanwhile, the size of SAR chips in the OpenSARShip dataset is mainly concentrated in 100 × 100 to 150 × 150. Refer to the universal practice described in [20,22], we resize the SAR images to 128 × 128 by CenterCrop function in torchvision transforms toolkit of Pytorch (one of the most popular deep learning frameworks). If the image size is smaller than 128 × 128, it was cropped, if otherwise, it was expanded. The exceeded parts are filled with pixel dots with a gray-value of 0. The targets of OpenSARShip datasets are in the center of the images, so we do not change the distribution of the targets in the images by the center crop or expansion.

Xavier [53] is a widely used initialization method in CNNs. Its basic design principle is to make the information flow better in the network. The variance of activation value and gradient of each layer should be kept as constant as possible. Refer to many state of the art CNNs (e.g., ResNet [9], DenseNet [10], MobileNets [35] etc.), we also adopt Xavier as the initialization method. We train the network by using mini-batch SGD, with an initial learning rate of 0.01 and a reducing factor of 0.1 after 30 epoches. The momentum parameter is set to be 0.9 and the weight decay parameter 0.0001. The number of iterations in training is 50, and the batch size is set to 30.

Experiments are carried out in the 64-bit Ubuntu 14.04 system. The software is mainly based on deep learning architecture of Pytorch and python development environment Pycharm. The hardware is based on an Intel (R) Core (TM) i7-6770K @ 4.00GHz CPU and two NVIDIA GTX1080 GPUs, with CUDA8.0 accelerating calculation.

### 3.3. Classification Experiment on MSTAR

In order to test the performance of our network in SAR image recognition, we conducted a classification experiment based on the MSTAR dataset, and selected four CNN models with good performance in SARA-ATR or CV field, namely Network-1, proposed by Wilmanski et al [23]. Network-2, A-ConvNets proposed by Chen et al. in literature [22], ResNet18 [9], and SE-ResNet50 [11].

**Figure 6:**
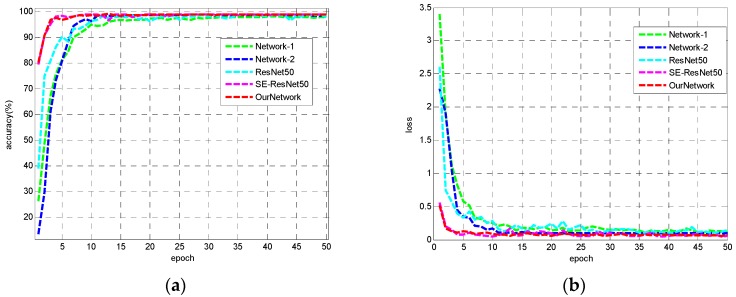
Training curves of 5 CNN models (**a**) accuracy curves (**b**) loss curves.

Figure 6 shows the training accuracy and loss curves of 5 models. It can be seen that due to the small amount of data in the MSTAR data set, the 5 CNN models can basically converge after 10 epoches, and all the networks can finally get close to 100% recognition accuracy on the training set. Our lightweight CNN and SE-ResNet50 have similar performance on the training set, and both of them converge faster than other models.

We define the accuracy of recognition *P* as the ratio of the number of samples correctly recognized to the total number of samples in the testing set, and use *P* as an indicator to evaluate the classification results. Table 8 shows the recognition accuracy on testing set, model size and total iteration times (total time spent on 50 training epoches) of five CNNs. 

Our network obtained the highest recognition accuracy of 99.54%, compared with Network-1, Network-2 and ResNet50. The recognition precision of our lightweight network and SE-ResNet50 is higher, which justify the fact that the introduction of visual attention mechanism can significantly enhance the ability of feature learning of CNN models. While achieving a slightly higher recognition accuracy than SE-ResNet50, our lightweight network has an obvious advantage in terms of iteration time and model size. The model size is about 1/5 of SE-ResNet50 and the iteration time is about 1/4 of it. According to the information in Table 8, our lightweight network has achieved better results in recognition accuracy and recognition efficiency. 

Table 9 shows the confusion matrix for the classification results of our lightweight network. As can be seen from the confusion matrix, each class has obtained an ideal accuracy, with a minimum recognition accuracy of 98.9% (BRDM-2) and maximum of 100% (T72, ZSU-131).

**Figure 7:**
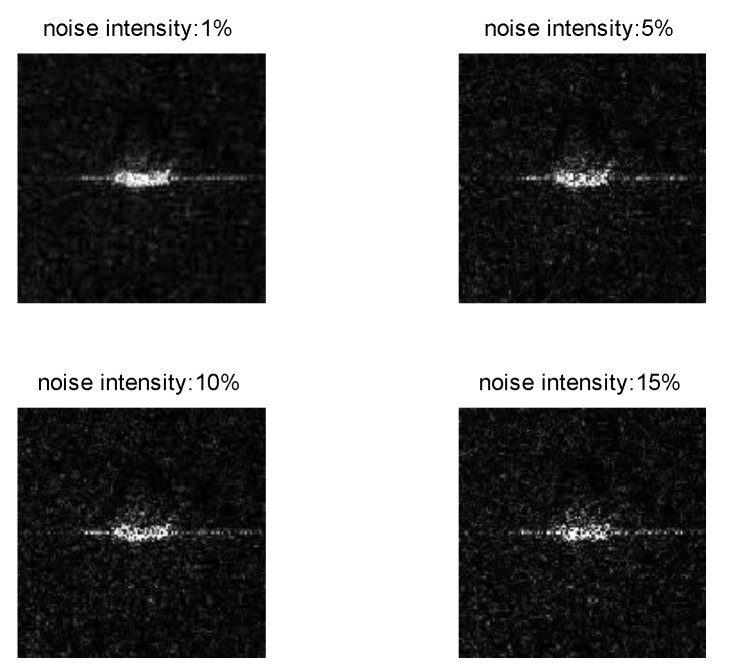
SAR images with different intensities of noise.

**Figure 8:**
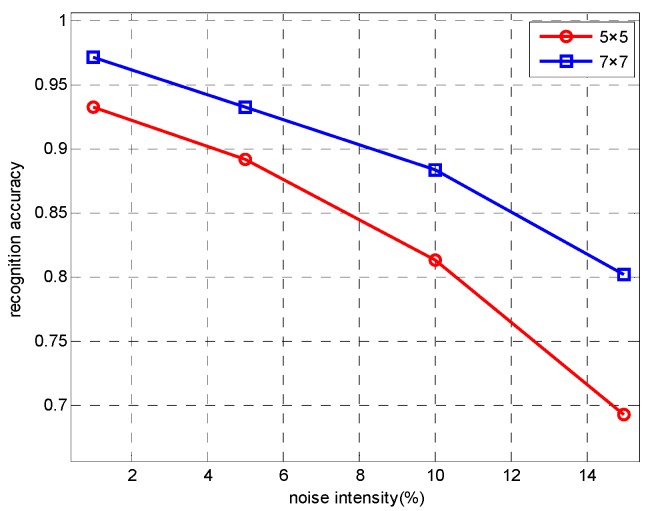
Recognition accuracy of different noise intensities.

An important characteristic of SAR images is often accompanied by the effects of noise. In order to test the anti-noise ability of our network, referring to the experimental methods in literature [22,54], we add different intensities noise obeying gamma distribution [55] in the SAR image by controlling the proportion of noise pixels in the whole image pixels. First, we design a noise function to generate random noise that obeying gamma distribution Ga(α,β), where α=1, β = 0.1. Then, randomly select a certain proportion of pixels in the test images and replace their values with independent and identically distributed samples generated by noise function. Finally, under different noise intensity, the proposed lightweight network is used to make a contrast experiment by changing the size of the convolution kernel in the first convolution layer. Examples of images with different intensities of noise are shown in Figure 7, and the experimental results are shown in Figure 8 and Table 10.

It can be seen from Figure 8 and Table 10 that with the increase of the noise intensity, the recognition accuracy of the 5 × 5 convolution kernel and the 7 × 7 convolution kernel decreases obviously. The recognition accuracy of 7 × 7 convolution kernel at any noise intensity is higher than that of 5 × 5 convolution kernel, which shows that the use of 7 × 7 convolution kernel has a better adaptability to noise. It is worth noting that when the noise intensity increases from 10% to 15%, the reduction of the recognition accuracy of the 5 × 5 convolution kernel is obviously greater than the 7 × 7 convolution kernel. So we can infer that the feature extraction ability of the small convolution kernel will be greatly affected under the high intensity noise condition. Based on the above experimental results, we choose the 7 × 7 convolution kernel when designing the first convolution layer of the network.

### 3.4. Classification Experiment on OpenSARShip

There is a serious data imbalance problem in the OpenSARShip data set. The study in [47] shows that random over-sampling and under-sampling are two good methods solving data imbalance problem in CNNs. In order to compare the processing capabilities for imbalanced data of random over-sampling, under-sampling and the WDM loss function mentioned in Section 2.3, we design five groups of ablation experiments based on the proposed lightweight network. The experimental conditions are shown in Table 11.

The over-sampling in Table 11 refers to random copying of minority classes, and eventually the number of samples in minority classes is the same as that of the majority classes. In the GRD mode, we randomly copy minority samples and finally make the number of training samples for each class to be 1600. Under-sampling randomly removes samples of majority classes to balance the number of samples in minority classes. However, because the number of samples of minority classes in OpenSARShip is too small (as shown in Table 4, there are only 45 training samples and 18 test samples in the tug class under GRD mode), the exclusive utilization of under-sampling will cause the number of samples too small to constitute effective training and testing set. Therefore, we take a compromise in group 3, under-sampling is used of the majority classes while over-sampling is used of the minority classes, and finally the number of samples in every class reached 500. The first three groups adopt the cross entropy loss function, the difference is whether the data is preprocessed. Group 4 adopts the WDM loss function we proposed, and the data is not preprocessed. Group 5 can be seen as a combination of group 3 and group 4, the WDM loss function is used on the basis of data processing.

**Figure 9:**
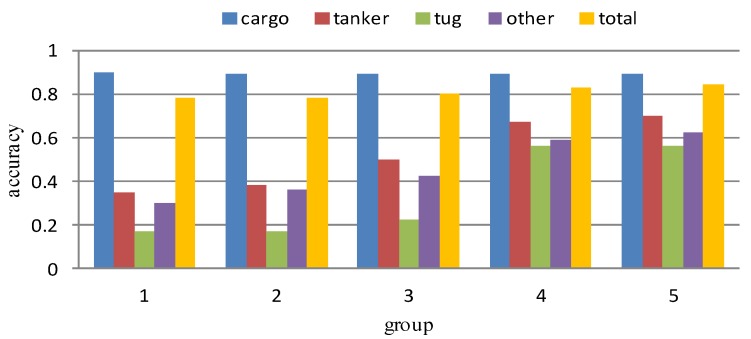
Result statistics of ablation experiments.

The results of prior classification experiments in Section 3.1.2 show that the recognition accuracy is not sensitive to different polarizations and imaging modes SAR image. So only the experimental results in the GRD mode with VH polarization are given here, as shown in Table 12, Table 13, Table 14, Table 15 and Table 16. The classification results of the five groups are summarized in Figure 9.

It can be seen from Table 12 and Figure 9 that group 1 obtains 78% of the overall recognition accuracy as the experimental benchmark, but the recognition rate of the four types of samples show a big difference. The recognition accuracy of majority class cargo reaches 90%, but the recognition accuracy of a minority class tug is only 17%, and the recognition accuracy of minority classes tanker and others is also at a lower level. In the experiment results of group 2 and group 3, the recognition accuracy of the minority classes is improved because of the use of over-sampling or the combination of over-sampling and under-sampling, but the recognition rate of tug is still very low (in the experiment of group 3, through data processing, the recognition accuracy can only be raised from 17% to 22%). The experiment results of group 4 shows that by using the WDM loss function, the recognition accuracy of the minority classes has greatly improved, with the recognition accuracy gap between cargo class and other minority classes obviously narrowed. The overall recognition accuracy reaches 83%, indicating that the WDM loss function effectively improves the adverse effects of imbalanced data on the recognition results. In group 5, we combine the data preprocessing method with the WDM loss function, and the recognition accuracy is slightly higher than that of group 4. It shows that the combination of the data level method and the algorithm level method will be a good way to solve the problem of data imbalance. In general, despite that the total classification accuracy from group 1 to group 5 slightly differs, the recognition accuracy of minority classes has greatly improved. It shows that the changes in the recognition accuracy of the minority classes are difficult to affect the overall recognition accuracy, only using the total recognition accuracy cannot accurately evaluate the recognition results.

### 3.5. Hyper-Parameters Experiment

In this section, we explain how to select the key hyper-parameters in the network. In Section 2.1, reduction ratio r is a variable parameter in channel attention bypass, and it represents the degree of compression of features on the channel. In order to get a suitable parameter, we use different r values to carry out recognition experiments on MSTAR dataset. The network used is the lightweight CNN proposed in this paper. In each comparison experiment, except for the value of r, the other conditions are the same. The results of the experiment are shown in Table 17.

The comparison in Table 17 reveals that with the increase of r, both accuracy and model size show a nonlinear downward trend. When r is increased from 8 to 16 and 32, the magnitude of the decrease in accuracy is significantly increased, so r is not as big as possible, the larger r can effectively compress the model size, but the over compression may also lead to the loss of information, and the decrease of the recognition accuracy. We found that when r = 8, a good tradeoff between accuracy and complexity is achieved, so we use this value for all experiments.

In Section 2.3, *m* is a hyper-parameter of the inter class loss *L*_2_, which is a limitation of distance between classes. In order to research its influence of the recognition accuracy, we conduct a series of comparative experiments under the same experimental environment of group 4 in Section 3.4. Experimental result is shown in Table 18.

We can conclude that the recognition accuracy is insensitive to *m*, when *m* is set to $2\times 10^{4}$, recognition accuracy of each class and entirety is more ideal. *m* represents a limitation of interclass distance and the bigger *m* brings the greater penalty. So we could also find that when $m>2\times 10^{4}$, the recognition accuracy is better than $m<2\times 10^{4}$.

In Section 2.4.1, we use the expansion factor *t* to control the data dimension, *t* is a coefficient. When *t* is less than 1, the data dimension is compressed, conversely, the data dimension is expanded. In residual structure, *t* is generally less than 1, while in the inverted residual structure, *t* is an integer greater than 1.

We set different *t* values for comparison experiments on the MSTAR dataset. The network used is the lightweight CNN proposed in this paper. In each comparison experiment, except for the value of *t*, the other conditions are the same. The results of the experiment are shown in the Table 19.

From Table 19, we can see that with the increase of *t*, the accuracy and the model size are increasing. When *t* is set to be 2 and 4, although it has a smaller model size, the accuracy rate is lower. When *t* is assigned to be 10, compared to 6, the accuracy rate is only a little higher, but model size has increased a lot, so we think 6 is the optimal value.

## 4. Conclusions and Future Work

This paper first designed a lightweight CNN based on visual attention and depthwise separable convolution for SAR image target classification. Then a new WDM loss function is proposed to solve the problem of data imbalance in data sets. Finally, a series of recognition experiments based on two open datasets of MSTAR and OpenSARShip are implemented. The experiment results on MSTAR show that compared with CNN model without visual attention mechanism (e.g., ResNet [9], Network in literature [23] and A-ConvNet [22]), our network achieves higher recognition accuracy, which indicate that the introduction of visual attention mechanism enhances the representation ability of CNN. Meanwhile, the model size and iteration time of our network is greatly reduced by the utilization of depthwise separable convolution. The ablation experiments on the OpenSARShip dataset compare the ability of several methods to handle imbalanced data. Experimental results indicate that the combination of resampling method and the WDM loss function can better weaken the impact of data imbalance on the recognition results. Nevertheless, there are still some limitations and shortcomings in our work.

1. The method we adopted in the paper belongs to supervised learning in machine learning field. The deep network needs a large number of data to train the parameters adequately, which restricts its application to a certain extent.
2. Our network needs the same size images as input, if the size of the input images is quite different, the recognition result will be affected. This problem can be solved by introducing space pyramid pooling (SPP) [56], which will be our future research direction.
3. The experimental results in Section 3.4 show that our network is somewhat sensitive to noise, and there is still much room for improvement in this aspect.

Last but not the least, weak supervised or unsupervised machine learning algorithm is an important development direction in the field of artificial intelligence. This kind of algorithm reduces the dependence on the training data to a certain extent, and makes the recognition process more intelligent. It is a worthwhile direction to introduce this algorithm into the field of SAR image and we think it will effectively enhance the intelligence and generalization ability of the recognition algorithm.

## Figures and Tables

**Table 1:** Data processing in the bottleneck structure.

Input	Operator	Output
h×w×k	1×1 conv	h×w×(tk)
h×w×(tk)	3×3 conv	$\frac{h}{s}\times \frac{w}{s}\times (tk)$
$\frac{h}{s}\times \frac{w}{s}\times (tk)$	1×1 conv	$\frac{h}{s}\times \frac{w}{s}\times k^{\prime }$

**Table 2:** The main structure of ResNet50 and our network.

Output Size	ResNet50	Our Network	Output Size
$64^{2}\times 64$	conv,7×7,64,stride2	conv,7×7,16,stride2	$64^{2}\times 16$
$32^{2}\times 256$	maxpool,3×3,stride2		$32^{2}\times 24$
	$\left[\begin{array}{l} conv,1\times 1\times 64,64\\ conv,3\times 3\times 64,64\\ conv,1\times 1\times 64,256 \end{array}\right]\times 3$	$\left[\begin{array}{l} conv,1\times 1\times 16,96\\ conv,3\times 3\times 1,16\\ conv,1\times 1\times 16,96\\ conv,1\times 1\times 96,24\\ CSAblock \end{array}\right]\times 2$	
$16^{2}\times 512$	$\left[\begin{array}{l} conv,1\times 1\times 256,128\\ conv,3\times 3\times 128,128\\ conv,1\times 1\times 128,512 \end{array}\right]\times 4$	$\left[\begin{array}{l} conv,1\times 1\times 24,144\\ conv,3\times 3\times 1,24\\ conv,1\times 1\times 24,144\\ conv,1\times 1\times 144,32\\ CSAblock \end{array}\right]\times 3$	$16^{2}\times 32$
$8^{2}\times 1024$	$\left[\begin{array}{l} conv,1\times 1\times 512,256\\ conv,3\times 3\times 256,256\\ conv,1\times 1\times 256,1024 \end{array}\right]\times 6$	$\left[\begin{array}{l} conv,1\times 1\times 32,192\\ conv,3\times 3\times 1,32\\ conv,1\times 1\times 32,192\\ conv,1\times 1\times 192,96\\ CSAblock \end{array}\right]\times 4$	$8^{2}\times 96$
$4^{2}\times 2048$	$\left[\begin{array}{l} conv,1\times 1\times 1024,512\\ conv,3\times 3\times 512,512\\ conv,1\times 1\times 512,2048 \end{array}\right]\times 3$	$\left[\begin{array}{l} conv,1\times 1\times 96,576\\ conv,3\times 3\times 1,96\\ conv,1\times 1\times 96,576\\ conv,1\times 1\times 576,320\\ CSAblock \end{array}\right]\times 3$	$4^{2}\times 320$
1×1×N	GAP, Fully connection, Cross entropy loss function	GAP, Fully connection, WDM loss function	1×1×N

**Table 3:** Number of samples in training and testing set.

	T62	T72	BRDM-2	BTR-60	2S1	ZSU-234	ZIL-131	D7	SLICY	Total
Training	299	423	298	256	299	299	299	299	298	2770
Testing	273	275	274	195	274	274	274	274	274	2387

**Table 4:** List of Experimental Data (VH/VV polarization).

	Cargo		Tanker		Tug		Dredging		Other	
	training	testing	training	testing	training	testing	training	testing	training	testing
GRD	1659	710	242	103	45	18	–	–	138	66
SLC	1225	525	345	146	19	7	17	6	189	78

**Table 5:** Experimental results of GRD mode with VH polarization.

	Cargo	Tanker	Tug	Other	*P*
Cargo	638	31	38	3	0.90
Tanker	41	36	9	17	0.35
Tug	12	0	3	3	0.17
Other	34	10	2	20	0.30
Total					0.78

**Table 6:** Experimental results of GRD mode with VV polarization.

	Cargo	Tanker	Tug	Other	*P*
Cargo	629	31	45	5	0.89
Tanker	38	39	9	17	0.38
Tug	11	1	3	3	0.17
Other	33	10	1	22	0.33
Total					0.77

**Table 7:** Experimental results of SLC mode with VH polarization.

	Cargo	Tanker	Tug	Dredging	Other	*P*
Cargo	478	33	8	6	10	0.91
Tanker	32	87	7	9	11	0.60
Tug	2	1	2	1	1	0.29
Dredging	2	1	1	2	0	0.33
Other	29	6	9	7	27	0.35
Total						0.78

**Table 8:** Experimental results of recognition accuracy, model size and iteration time.

Networks	Network-1	Network-2	ResNet50	SE-ResNet 50	Our Network
*P* (%)	95.41	98.05	98.23	99.41	99.54
Model size (Mb)	19.2	21.6	98.5	112.6	24.2
Iteration time (s)	442	557	1259	1576	403

**Table 9:** Confusion matrix for the experimental results of lightweight network.

	T62	T72	BRDM-2	BTR-60	2S1	ZSU-234	ZIL-131	D7	SLICY	P
T62	272	0	0	1	0	0	0	0	0	99.64
T72	0	275	0	0	0	0	0	0	0	100
BRDM-2	0	2	271	0	0	0	0	1	0	98.90
BTR-60	0	0	0	193	0	1	1	0	0	98.97
2S1	0	0	0	0	273	0	1	0	0	99.64
ZSU-234	0	0	0	0	0	274	0	0	0	100
ZIL-131	0	1	0	0	0	0	273	0	0	99.64
D7	0	0	0	0	0	1	0	273	0	99.64
SLICY	0	2	0	0	0	0	0	0	272	99.27
Total										99.54

**Table 10:** Recognition accuracy of 5 × 5 and 7 × 7 kernel size.

Noise Intensity Kernel Size	1%	5%	10%	15%
5 × 5	0.9333	0.8921	0.8133	0.6928
7 × 7	0.9714	0.9326	0.8835	0.8019

**Table 11:** Setting of experimental conditions on OpenSARShip dataset.

	Over-Sampling	Under-Sampling	Cross Entropy Loss	WDM Loss
Group 1 (baseline)	×	×	√	×
Group 2	√	×	√	×
Group 3	√	√	√	×
Group 4	×	×	×	√
Group 5	√	√	×	√

**Table 12:** Experimental results of group 1 (baseline, same as Table 5).

	Cargo	Tanker	Tug	Other	*P*
Cargo	638	31	38	3	0.90
Tanker	41	36	9	17	0.35
Tug	12	0	3	3	0.17
Other	34	10	2	20	0.30
Total					0.78

**Table 13:** Experimental results of group 2.

	Cargo	Tanker	Tug	Other	*P*
Cargo	635	31	41	3	0.89
Tanker	38	39	9	17	0.38
Tug	12	0	3	3	0.17
Other	33	7	2	24	0.36
Total					0.78

**Table 14:** Experimental results of group 3.

	Cargo	Tanker	Tug	Other	*P*
Cargo	632	34	41	3	0.89
Tanker	30	51	7	15	0.50
Tug	11	0	4	3	0.22
Other	30	6	2	28	0.42
Total					0.80

**Table 15:** Experimental results of group 4.

	Cargo	Tanker	Tug	Other	*P*
Cargo	630	35	41	4	0.89
Tanker	22	69	5	7	0.67
Tug	5	0	10	3	0.56
Other	19	6	2	39	0.59
Total					0.83

**Table 16:** Experimental results of group 5.

	Cargo	Tanker	Tug	Other	*P*
Cargo	632	36	39	3	0.89
Tanker	20	72	5	6	0.70
Tug	5	0	10	3	0.56
Other	18	5	2	41	0.62
Total					0.84

**Table 17:** Experimental results of different value of r.

Reduction Ratio *r*	Accuracy (%)	Model Size (Mb)
4	99.58	31.6
8	99.54	24.2
16	99.16	21.9
32	98.08	18.5

**Table 18:** Recognition accuracy (P) of samples under different values of *m*.

	Cargo	Tanker	Tug	Other	Total
$m=1.5\times 10^{4}$	0.87	0.65	0.50	0.56	0.81
$m=1.8\times 10^{4}$	0.88	0.65	0.50	0.58	0.82
$m=2\times 10^{4}$	0.89	0.67	0.56	0.59	0.83
$m=2.5\times 10^{4}$	0.89	0.66	0.56	0.59	0.83
$m=3\times 10^{4}$	0.88	0.65	0.50	0.58	0.82

**Table 19:** Experimental results of different value of *t*.

Expansion Factor *t*	Accuracy (%)	Model Size (Mb)
2	95.62	17.6
4	96.37	21.5
6	99.53	24.2
10	99.56	31.1
